# Corrosion Sensor for Monitoring the Service Condition of Chloride-Contaminated Cement Mortar

**DOI:** 10.3390/s100404145

**Published:** 2010-04-26

**Authors:** Shuang Lu, Heng-Jing Ba

**Affiliations:** School of Civil Engineering, Harbin Institute of Technology, Harbin, 150006, China; E-Mail: bahengjing2001@yahoo.com.cn

**Keywords:** corrosion sensor, cement mortar resistance, corrosion rate, reference electrode, linear polarization resistance

## Abstract

A corrosion sensor for monitoring the corrosion state of cover mortar was developed. The sensor was tested in cement mortar, with and without the addition of chloride to simulate the adverse effects of chloride-contaminated environmental conditions on concrete structures. In brief, a linear polarization resistance method combined with an embeddable reference electrode was utilized to measure the polarization resistance (*R*_p_) using built-in sensor electrodes. Subsequently, electrochemical impedance spectroscopy in the frequency range of 1 kHz to 50 kHz was used to obtain the cement mortar resistance (*R*_s_). The results show that the polarization resistance is related to the chloride content and *R_s_*; ln (*R*_p_) is linearly related to the *R*_s_ values in mortar without added chloride. The relationships observed between the *R_p_* of the steel anodes and the resistance of the surrounding cement mortar measured by the corrosion sensor confirms that *R*_s_ can indicate the corrosion state of concrete structures.

## 1. Introduction

In recent years, chloride-induced corrosion of structural steel has caused serious damage to concrete structures all over the world. A large number of harbor bridges, dams, docks and harbor structures have been damaged by chloride penetrating from the surrounding environment, especially in tidal zones and coastal areas [1,2]. The premature failure of coastal concrete structures often causes financial losses that are much higher than the initial construction cost [3], and it has been estimated that these failures account for more than 35% of the total amount of construction work in Europe.

In addition to ameliorating the above economic concerns, *in situ* corrosion sensors might provide information about service conditions and inform further design requirements. This new branch of sensor technology is largely based on the development of novel electrochemical monitoring techniques, including half-cell potential, linear polarization resistance (LPR), AC impedance spectroscopy, electrical resistance measurements and several other techniques [4–6]. Each of these sensors or techniques has advantages and disadvantages that determine the environment in which it is used [7–10]. The LPR method may be the most reliable and valuable technique for addressing the intrusion of chloride in coastal concrete structures, as it allows for *in situ* assessment of service conditions in chloride-contaminated concrete [11].

Several types of linear polarization sensors have been developed to improve the accuracy of LPR methods. One well-known apparatus, the guard-ring system, was developed to confine the excitation current within a defined area [12]. Unfortunately, this system is less precise than unguarded electrode devices; it has been shown to underestimate the amount of metal loss by a factor of 4–6 [13]. Andrade and Martinez presented a calibration of LPR measurements using the modulated confinement of the current method (MCC), and compared the electrochemical results with gravimetric losses of rebar [14]. Although the polarization resistance (*R*_p_) measured in this manner was comparable to that obtained with the adopted gravimetric method, the above-mentioned MCC method has been shown to be less reliable than the normal LPR method in measuring the low resistance of concrete [15]. The polarization resistance, *R*_p_, is commonly used as a measure of metal’s resistance to corrosion damage. A high value of *R*_p_ is associated with high corrosion prevention ability; a low value of *R*_p_ indicates high potential corrosion activity [16].

The electrical resistance of cover-zone concrete is also related to the principal stages in the service life of a structure: the initiation period (chloride penetration) and the propagation period (corrosion rate) [9]. Although the concrete resistance does not determine whether steel is actively corroding in concrete, it can indirectly elevate the corrosion risk of steel embedded in cover-zone concrete. Non-destructive monitoring of the concrete resistance has frequently been mentioned as an important method of evaluating service conditions in chloride-contaminated concrete structures [8]. In light of the sensor electrode polarization induced by a direct current, most methods for cover resistance measurements use constant-frequency alternating current (AC) signals [17]. However, this method has been found to be not accurate enough, and the results are poorly reproducible if a constant frequency is adopted [18].

Consequently, an embeddable corrosion sensor for monitoring the comprehensive service conditions of chloride-contaminated concrete structures was developed in this study. First, the expanded LPR method combined with an embeddable Ti/MnO_2_ reference electrode was adopted for measuring the *R*_p_ of the built-in steel anodes [19]. Subsequently, following our recent work, electrochemical impedance spectroscopy with a frequency range of 1 kHz to 50 kHz was adopted to obtain a more precise electrical resistance value and account for the non-homogeneity of the cement mortar and the interfacial characteristics of the mortar and steel sensor anodes [20].

However, it is worth pointing out that the *R*_p_ of structural steel in service, rather than built-in anodes, should ultimately be measured or evaluated; only then will the results of the sensor system have any meaning. Unfortunately, the steel used in concrete structures usually has a complex structure (usually resulting from an unknown effective surface area) and is usually disturbed by interference current from either the ground or human actions. Thus, attempts to quantify the *R*_p_ of structural steel using traditional electrochemical technology have met both theoretical and empirical problems [13]. In order to overcome the limitations arising from imprecise methods, we propose a formula to statistically analyze the values obtained from the corrosion sensors. This paper summarizes the relationship between the cover-zone mortar resistance (*R*_s_) and the *R*_p_ of the steel anodes, and aims to predict *R*_p_ from the *R*_s_ values of the surrounding cover mortar using the parameters obtained from the developed sensors, instead of directly measuring the *R*_p_ of the structural steel.

## 2. Experimental

### 2.1. Materials

P·O 42.5 cement from Harbin Cement Co. LTD was used for all experiments in this study. River sand with a fineness modulus of 2.4 was used as a fine aggregate. The chemical composition of the steel anodes used for the sensor, corresponding to Q235 steel, is given in Table 1. Q235 steel is widely used in civil engineering and especially in coastal constructions in China.

### 2.2. Sensor Arrangements

**Figure 1.**
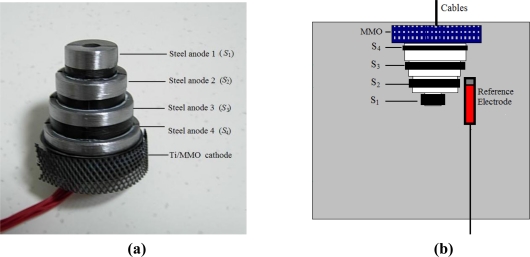
(a) Photo of the embeddable corrosion sensor. (b) Schematic of the placement of the corrosion sensor in cement mortar.

Figure 1 presents a photo and a detailed schematic of the corrosion sensor embedded in cement mortar. Each of the four differently-sized steel anode rings was fixed on a fabricated nylon tower [see Figure 1 (b)], spaced 10 mm from one another. The detailed geometrical design of each steel anode is given in Table 2. Cables connected to each single steel anode, *S*_1_∼*S*_4,_ were run out of the cover-zone mortar.

This geometrical sensor design ensures that:

1. Each steel anode has an equal exposure area of about 668 mm^2^. The surface area of the cathode (more than 3,000 mm^2^) is sufficiently larger than the anodes to reduce errors due to the lateral current distribution on the surface of the steel anodes [21].
2. The cement mortar cover over each steel anode is not affected by the parts of the sensor, thus, the penetration of chloride into each anode and the carbonation of the surrounding cement mortar are not influenced by the adjacent anodes.
3. The sensors can be used in existing concrete structures by inserting them into holes drilled in cover-zone concrete. Furthermore, all of the measured parameters are depth-related. In this way, the penetration of chloride and/or carbonation from the cement mortar surface into the cement mortar and the subsequent corrosion risk of the reinforcing structures can be measured immediately.

A cathode made of Ti/MMO (mixed metal oxide-coated titanium), which is widely used in the cathodic protection system, was placed 10 mm above anode *S*_4_. A fabricated long-term Ti/MnO_2_ reference electrode (RE) was embedded adjacent to the corrosion sensor for potential measurements, and the stable potential of the RE is −40 ± 5 mV referenced to the saturated calomel electrode (SCE) [18]. Each sensor was embedded in the middle of a cubic cement mortar specimen (10 cm × 10 cm × 10 cm). The distance between the upper end of the sensor and the upper surface of the cement mortar was 2 mm.

### 2.3. Specimens and Storage Conditions

#### 2.3.1. Mixture proportions of the cement mortar

To evaluate the electrical resistance and the polarization resistance measured by the sensor in chloride-contaminated cement mortar, different cement chloride contents were obtained by dissolving NaCl in water (see Table 3). Two specimens and sensors were used for each mixture proportion (*A* to *D*).

#### 2.3.2. Short-term wetting conditions

The cement mortar specimens were cured for 28 days at an ambient temperature of 20 ± 1 °C and a relative humidity (RH) of 95%. Afterwards, the specimens were vacuum-saturated, and then placed in a closed curing box at an ambient temperature of 20 ± 1 °C and an RH of 65% for measurements.

The above procedures were designed to eliminate the adverse influence of a moisture gradient in the cement mortar and to simulate a kind of “short-term wetting” condition. “Short-term wetting” characterizes any form of wetting (e.g., fog, sprayed water, splashed water or a rain shower) that does not cause oxygen deprivation at the steel surface [22].

### 2.4. Measurement Theory and Procedure

#### 2.4.1. Electrochemical impedance spectroscopy (EIS)

EIS tests were conducted at the rest potential in the frequency range of 1 kHz to 50 kHz using signal amplitude of 10 mV. The real part (Z′) and the imaginary part (Z″) of the sensor cell impedance were recorded.

**Figure 2.**
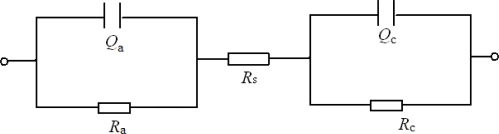
Equivalent circuit of the corrosion sensor embedded in the cement mortar.

It is worth noting that the charging of a discontinuous, inhomogeneous interface between the cement mortar and the steel anode leads to a CPE-like (Constant Phase-angle Element) response. A response of this type in the steel anode-mortar system is therefore to be expected, due to both the lack of surface homogeneity in the reinforcements and the eminently heterogeneous nature of mortar [23]. Consequently, the modified circuit shown in Figure 2 was used to quantitatively interpret the electrical signal response of the steel anode-mortar system.

The elements *C*_a_ and *C*_c_ (interfacial capacitance of the steel anode and the Ti/MMO cathode) are replaced by the constant phase elements *Q*_a_ and *Q*_c_. *R*_a_ (in Ω) and *R*_c_ represent the polarization resistances of the steel anode and cathode, respectively. *R*_s_ represents the cement mortar resistance between the steel anode and cathode. The symbol *Q* usually denotes a CPE element, and the impedance of *Q* can be given as follows:

(1) $$Z=\frac{1}{Y_{0}}\times {\left(jw\right)}^{-n}.$$

Where n is a constant, *Y*_0_ (in Ω^−1^·cm^−2^·s^−n^) is a parameter derived from the capacitance *C* (in F), and *w* is the frequency. In light of the interfacial properties of the built-in electrodes in the previously described sensor system, the impedance can be simplified, according to Figure 2, and written as follows [24]:

(2) $$Z^{\prime }=\frac{\frac{1}{R_{a}}+Y_{0}w^{n}\text{cos}(\frac{n\pi }{2})}{{\left(\frac{1}{R_{a}}\right)}^{2}+(\frac{2}{R_{a}})Y_{0}w^{n}\text{cos}(\frac{n\pi }{2})+{\left(Y_{0}w^{n}\right)}^{2}}+\frac{\frac{1}{R_{c}}+Y_{0}w^{n}\text{cos}(\frac{n\pi }{2})}{{\left(\frac{1}{R_{c}}\right)}^{2}+(\frac{2}{R_{c}})Y_{0}w^{n}\text{cos}(\frac{n\pi }{2})+{\left(Y_{0}w^{n}\right)}^{2}}+R_{s}.$$

(3) $$Z^{\prime \prime }=\frac{Y_{0}w^{n}\text{sin}(\frac{n\pi }{2})}{{\left(\frac{1}{R_{a}}\right)}^{2}+(\frac{2}{R_{a}})Y_{0}w^{n}\text{cos}(\frac{n\pi }{2})+{\left(Y_{0}w^{n}\right)}^{2}}+\frac{Y_{0}w^{n}\text{sin}(\frac{n\pi }{2})}{{\left(\frac{1}{R_{c}}\right)}^{2}+(\frac{2}{R_{c}})Y_{0}w^{n}\text{cos}(\frac{n\pi }{2})+{\left(Y_{0}w^{n}\right)}^{2}}.$$

#### 2.4.2. Linear polarization resistance

The polarization resistance of the electrodes was determined in each of the cement mortar samples. The potential was swept at a scan rate of 0.2 mV/s, from −20 to 20 mV referenced against the free corrosion potential of the steel anode. Measurement configurations with three electrodes were used, with each steel anode acting as a working electrode, the embedded RE acting as a reference and the Ti/MMO cathode in the mortar acting as a counter electrode. The polarization resistance *R*_p_ (in Ω) can be deduced from the response Δ*I* (in A·cm^−2^) of the steel anodes to a small amplitude step of potential Δ*E* (in V):

(4) $$R_{p}=\Delta E/\Delta I_{\left(\Delta E\to 0\right)}$$

## 3. Results and Discussion

### 3.1. Cover-zone Cement Mortar Resistance

**Figure 3.**
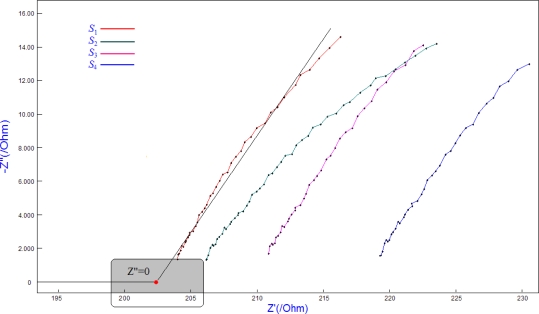
Nyquist plots of Z′ *versus* Z″ for the sensor system embedded in 3% sodium chloride mortar (mix *C*) obtained at various frequencies (1 kHz to 50 kHz).

Figure 3 presents the impedances of each steel anode (*S*_1_–*S*_4_) in the 3% sodium chloride mortar sample *C*. The diagrams obtained from the different steel anodes are similar in shape to each other. This type of spectrum is usually interpreted by using the model described above, consisting of a CPE (*Q*_a_ and *Q*_c_) in parallel with the polarization resistance (*R*_a_ and *R*_c_), in addition to the cement mortar resistance (*R*_p_). The adopted frequencies (1 kHz to 50 kHz) are sufficiently high that $Y_{0}w_{n}\text{cos}(\frac{n\pi }{2})$ is much greater than $\frac{1}{R_{a}}$ or $\frac{1}{R_{c}}$ in Equations (2) and (3). The impedance curve of the sensor system in the cement mortar exhibits a rectilinear regulation trend (see Figure 3). Consequently, the relationship between *Z*′ and *Z*″ can be simplified and is given as follows:

(5) $$Z^{\prime \prime }=aZ^{\prime }+b.$$

where *a* represents the slope, and *b* represents the intercept of the impedance curve. The values *a* and *b* can be calculated from the data shown in Figure 3. Then, *R*_s_ (the red point in the shaded area, corresponding to *Z*″ = 0) can be obtained.

Most of the existing works on alternating current (AC) approaches to resistance measurements only measure the impedance behavior at a fixed frequency. Generally, the traditional measured results are not exactly the true resistance, due to interference with the interface capacitance between the electrodes and the cement mortar [17]. The adverse effects of the interface capacitance are generally reduced by using the present method.

**Figure 4.**
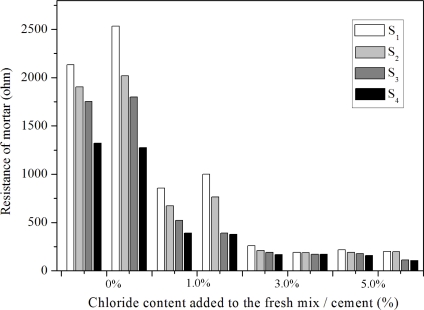
The resistance values of cement mortars with different chloride contents (*A* to *D*).

Figure 4 shows the depth-related resistance between the different anodes (*S*_1_–*S*_4_) and a cathode embedded in different cement mortars (*A* to *D*). Mix *A*, with no added chloride, was investigated as a reference mortar. Each sample was measured six times to give an average resistance value for each electrode pair with very low scatter. The resistance values for this method, presented in Figure 4, represent the measurements from two separate samples. Error range of ± 5% was observed when comparing the resistance of a solution with known conductivity (0.1 mol/L NaCl, 0.011644 S·cm^−1^) with the calculated resistance value [18].

It is shown in Figure 4 that the resistance is proportional to the chloride content and increases with increasing cover depth. As expected, it was found that the cement mortar without added chloride exhibited a very high resistance, above 1250 ohms. In contrast, a lower mortar resistance (less than 1,000 ohms) is measured in the mortar samples with 1–5% chloride in the mixture. Moreover, the addition of 1% chloride to the cement mortar produces a sharp decrease in the mortar resistance. A difference factor of more than 10 was observed when the resistance values of specimens *C* and *D* were compared to the values observed in specimen *A*. Because the cement mortar has a certain microstructure and chemical composition, the resistance of concrete structures depends entirely on the environmental humidity, temperature and concentration of penetrating ions, *etc.* [25]. The decrease in mortar resistance with increasing chloride content can be explained by the chloride ion exchange interaction, but this seems to be insignificant when the chloride content is greater than 3% (see the resistance values of mixtures *C* and *D* shown in Figure 4).

The relationship between mortar resistance and corrosion rates is still a subject of study. However, measurements of corrosion rates as a function of chloride penetration into the mortar have shown that the mortar resistance has a significant effect on the corrosion rate, especially in the case of mortars with high moisture content [26]. This strongly suggests that, for the investigated cement mortar and environmental factors, measured resistance values could be used to differentiate between the passive and active states of the surrounding steel reinforcements. However, such a measurement would be cumbersome, and in this work the polarization resistance (*R*_p_) was measured and analyzed as discussed in the next section.

### 3.2. Polarization Resistance Measured by the LPR Method

**Figure 5.**
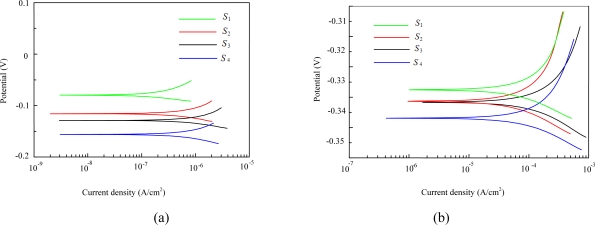
Polarization curves (*E*-log *i*) of the built-in steel anodes (*S*_1_–*S*_4_) of the sensor embedded (a) in cement mortar *A*; (b) in chloride-contaminated mortar (sample *C*).

The LPR test results for each steel anode (*S*_1_ to *S*_4_) in mortars *A* and *C* are shown in Figure 5. Duplicate experiments provided essentially the same results. Figure 5 shows that the difference between the corrosion potential of *S*_1_ and *S*_4_ is approximately 100 mV in mortar *A*. The recorded corrosion potential tends to increase with depth; this result may be explained by the availability of oxygen at various depths. However, such deviation seems not to be significant in mortar *C* due to its poor cover-zone quality.

In addition, corrosion potential is a clear indicator of cover mortar quality. A more positive corrosion potential is usually related to a lower corrosion rate in steel [19]. The steel anodes embedded in mix *A*, depicted in Figure 5 (a), exhibit a shift in the corrosion potential towards a more positive value, indicating the ability to passivate. In contrast, the steel anodes embedded in mix *C* shift to more negative potential values, less than −300 mV, due to the high chloride content near the surface of the steel anodes. It is found that *S*_4_, which is near the cement mortar surface, showed the lowest corrosion potential, below −340 mV in mix *C* (*versus* the RE), which may indicate a higher corrosion risk according to ASTM C 876–91.

The direction of the shift in the polarization curve is another immediate descriptor of the quality of the steel anode surface. As shown in Figure 5 (a), the steel anodes exhibited a negligible current in sample *A*, further indicating a tendency towards the passive state. Generally, the polarization curve for the steel anodes in mortar *A* moved towards more positive potentials (higher) as the cover depth increased. In contrast, a nearly opposite trend was observed in the polarization curves of each steel anode in mix *C*. An increase in current density with increasing electrode potential was observed in both the cathodic and anodic polarization curves. This indicates that the anodic current densities were high enough to guarantee that the steel anodes underwent significant corrosion.

**Figure 6.**
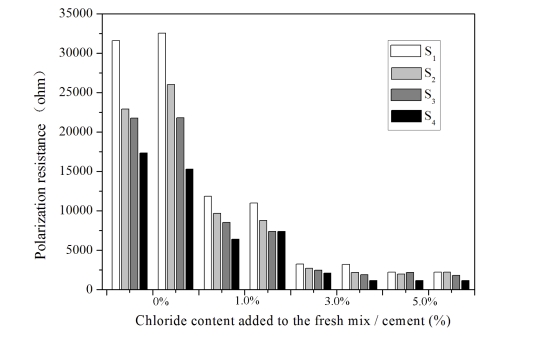
*R*_p_ results from LPR tests of corrosion sensors in cement mortar samples with different chloride contents (*A* to *D*).

Figures corresponding to mixes *B* and *D* (not shown here) show that the steel anodes exhibited similar curves in all three of the chloride-contaminated mortar samples. Previous results, such as polarization curves and corrosion potentials, dealt only with a simplistic prediction of the corrosion risk and a qualitative analysis of the corrosion tendency. For quantitative purposes, a detailed and precise prediction is necessary. The results shown above can be improved by using the *R*_p_ values obtained from Equation (4). Figure 6 depicts the *R*_p_ values corresponding to the LPR curves obtained from the embedded sensors in all of the cement mortar samples (*A* to *D*).

Figure 6 shows that the resistance values measured by the corrosion sensors in each mortar sample follow a descending trend of *A*>*B*>*C*>*D*, similar to the trend of polarization resistance depicted in Figure 4. In addition, the *R*_p_ values recorded by the sensors embedded in the mortar without chloride are two to 10 times higher than the values of the chloride-contaminated samples. This agrees with results presented in prior investigations, obtained from marine concrete structures with similar w/c ratios [27]. Thus, the cement mortar corrosion risk can be estimated to some extent by measuring *R*_p_.

It should be pointed out that the *R*_p_ values are higher in *S*_1_ and lower in *S*_4_ than those corresponding to the other anodes embedded in the same mortar. This finding is probably due to the depth-related oxygen availability in cement mortar because linear correlations have been established between the distance from the surface and O_2_ concentrations [28]. It is known that the cathodic reaction responsible for the corrosion of steel in concrete requires a certain amount of oxygen. It can only be assumed that, according to what has been previously described, the protective mortar layer surrounding the steel surface plays a decisive role in the corrosion of the steel anode. This assumption can be explained by the lower oxygen availability near *S*_1_, which increases *R*_p_, even when 3% or 5% chloride was added (see Figure 6).

**Figure 7.**
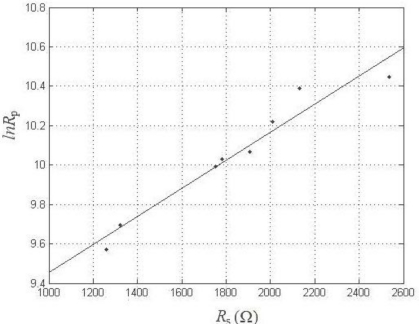
ln *R*_p_ *versus R*_s_ plot.

Figure 7 presents a plot of ln *R*_p_ *versus R*_s_ in the mortar without added chloride, in order to demonstrate the relationship between these two parameters. A nearly linear relationship between ln *R*_p_ and *R*_s_ was observed. The straight line in Figure 7 is described by the following equation:

(6) $$\text{ln}\;R_{p}=0.000716\times R_{s}+8.7372\;\;\;\;\;\;\;\;r^{2}=92.5\%$$

The linear relationship shows that, in the absence of chloride contamination, *R*_p_ increases as the mortar resistance increases. However, in this case, the corrosion rate is mainly controlled by the oxygen supply and qualities of the cement mortar. By assuming that all of the electrodes and built-in units of the corrosion sensors were operating at the same temperature and relative humidity, the mortar can be treated as a quantifiable aggressive environment. Thus, this study further confirms that both parameters (*R*_p_ and *R*_s_) reflect the corrosion state of the cement mortar, regardless of the current amount of corrosion [29]. This relationship provides the convenience for evaluating the corrosion state of cover-zone concrete which is based on the cover mortar resistance, instead of being determined by *R*_p_ which is difficult to obtain on-site. It offers a simple solution, in a limited domain (mortar without chloride contamination), for a complex problem. However, it is apparent that in order to apply this relationship to deduce the *R*_p_ values of structural steel in service, we must pay attention to not only the complicated exposure circumstances, but also to the heterogeneity of the concrete due to the addition of the coarse aggregate.

In addition, to determinate the chloride contents using a new developed chloride ions sensor, and to clarify the relationship among the chloride contents, the cover-zone concrete resistance and the polarization resistance, further studies are now in process. Some adjustment is needed to use this relationship to estimate the corrosion state of field concrete structures in which chloride ions diffuse into the concrete. Another point worth emphasizing is that this model was developed at a given constant temperature and relative humidity. More research is required, for example, studying this relationship with temperature sensors, humidity sensors [30,31], chloride content sensors, *etc.*, before the final goal of *R*_p_ prediction can be achieved.

## 4. Conclusions

In this work, a sensor system consisting of a reference electrode, an electrical resistance measurement unit and a polarization resistance unit was developed and tested in cement mortar, with and without chloride added, to simulate the corrosion behavior of chloride-contaminated concrete structures.

To account for the CPE-like characteristics of the interface between the cement mortar and steel anodes, a range of frequencies from 1 kHz to 50 kHz was used to obtain more precise *R*_s_ values. When the measured *R*_s_ values for the mortar cover with or without added chloride are compared, the *R*_s_ value of the chloride-containing mortar is found to always be much lower. The addition of chloride increases the electrolytic conductivity of the mortar and may cause a potential increase in the corrosion risk. The mortar resistance depends significantly on the chloride content of the mortar, but this effect does not seem to be significant when the chloride content is greater than 3%.

The LPR method, combined with an embeddable reference electrode adjacent to the steel anode, was adopted to measure the *R*_p_ value of the steel anodes built into the sensor. The polarization resistance depends strongly on the mass ratio of the admixed chloride and on the oxygen availability, which depends on the thickness of the mortar cover. Thus, the electrical resistance of the mortar cover is proven to be an effective parameter for evaluating the corrosion risk of steel anodes, independently of mix design or exposure conditions. The good quality mortar sample presented a value of *R*_p_ ≈ 1.5 × 10^4^ Ω while the mortar samples with added chloride presented values below 1.0 × 10^4^ Ω.

To determine the corrosion rate of structural steel, a predictive relationship for characteriing *R*_p_ from the obtained mortar cover resistance is presented for the cement mortar without added chloride. To allow the corrosion rate to be estimated in realistic concrete structures, the relationships among *R*_s_, *R*_p_ and the free chloride content are currently under study using the developed corrosion sensor and an external chloride content sensor to simultaneously measure *R*_p_ and the free chloride content.

## Figures and Tables

**Table 1.** Chemical composition of the steel anodes used in this study.

Elements in %	C	Mn	P	S	Si	Fe
Steel anode	0.181	0.580	0.012	0.023	0.350	97.5

**Table 2.** The geometrical sizes of the steel anode rings used in this study (mm).

Anode	Inside diameter	Outside diameter	Depth
*S*_1_	8.0	24.0	8.7
*S*_2_	24.0	33.0	7.7
*S*_3_	33.0	40.0	6.7
*S*_4_	40.0	46.0	6.0

**Table 3.** Mixture proportions of the cement mortar specimens used in this study.

Mix	Cement	Fine agg.	Water	NaCl (%)
*A*	1	3	0.40	0
*B*	1	3	0.40	1.0
*C*	1	3	0.40	3.0
*D*	1	3	0.40	5.0
